# Social prescribing for individuals with mental health problems: a qualitative study of barriers and enablers experienced by general practitioners

**DOI:** 10.1186/s12875-020-01264-0

**Published:** 2020-09-21

**Authors:** Henry Aughterson, Louise Baxter, Daisy Fancourt

**Affiliations:** Department of Behavioural Science and Health, Institute of Epidemiology, University College London (UCL), 1-19 Torrington Place, London, W1CE 7HB UK

**Keywords:** Social prescribing, General practice (GP), Community engagement, Community, Mental health, COM-B, Theoretical domains framework (TDF), GP behaviour, Behaviour(al) change theory, United Kingdom (UK)

## Abstract

**Background:**

There is growing evidence for the use of social prescribing as a means to improve the mental health of patients. However, there are gaps in understanding the barriers and enablers faced by General Practitioners (GPs) when engaging in social prescribing for patients with mental health problems.

**Methods:**

This study uses a qualitative approach involving one-to-one interviews with GPs from across the UK. The COM-B model was used to elucidate barriers and enablers, and the Theoretical Domains Framework (TDF) and a Behaviour Change Theory and Techniques tool was used to identify interventions that could address these.

**Results:**

GPs recognised the utility of social prescribing in addressing the high levels of psychosocial need they saw in their patient population, and expressed the need to de-medicalise certain patient problems. GPs were driven by a desire to help patients, and so they benefited from regular positive feedback to reinforce the value of their social prescribing referrals. They also discussed the importance of developing more robust evidence on social prescribing, but acknowledged the challenges of conducting rigorous research in community settings. GPs lacked the capacity, and formal training, to effectively engage with community groups for patients with mental health problems. Link workers, when available to GPs, were of fundamental importance in bridging the gap between the GP and community. The formation of trusting relationships was crucial at different points of the social prescribing pathway, with patients needing to trust GPs in order for them to agree to see a link worker or attend a community activity, and GPs requiring a range of strong inter-personal skills in order to gain patients’ trust and motivate them.

**Conclusion:**

This study elucidates the barriers and enablers to social prescribing for patients with mental health problems, from the perspectives of GPs. Recommended interventions include a more systematic feedback structure for GPs and more formal training around social prescribing and developing the relevant inter-personal skills. This study provides insight for GPs and other practice staff, commissioners, managers, providers and community groups, to help design and deliver future social prescribing services.

## Background

There is growing recognition internationally of the limits of biomedically-centred approaches to tackling many of the leading health problems. It is estimated that 1 in 6 adults experience a common mental health disorder such as anxiety, depression or obsessive-compulsive disorder [1, 2]. Multiple factors underlying these high rates have been suggested, including increasing inequality and economic uncertainty, the rise of chronic physical illness and obesity, cultural individualism, increasing levels of loneliness and an ageing population [3–6]. Moreover, obesity and chronic physical health conditions are also significantly influenced by one’s mental health and social circumstances [7].

First-line approaches in the UK for treating the common mental health disorders consist of medication use such as anti-depressants, and psychological therapies. A meta-analysis of anti-depressant use has shown significant effects of the drugs compared with placebo in severe depression, but that the effect in mild or moderate depression may be “minimal or non-existent” [8]. Cognitive behavioural therapy (CBT), the most common form of psychological therapy in the UK, can be an effective treatment, however is normally only available to the person for 8–12 weeks, often with long waiting times. Further, a meta-analysis found its efficacy as a treatment for depression has been diminishing over time [9].

There has been rising support in recent years for approaches that support people’s mental health in ways other than medication and time-bound psychological therapy (e.g. IAPT-accessed CBT). Academics and practitioners have called for more community-based approaches that are personalised to an individual’s circumstances, available longer term, and address the social determinants of mental health [10, 11]. Social prescribing is potentially one such approach, defined as the referral of patients, often from a GP (General Practitioner), to sources of support within their community such as walking groups, Men’s sheds, singing groups, lunch clubs, arts activities and community gardening. This can occur via a link worker, who sits between the GP and community groups, and works with patients to discuss and agree their “social prescription”. Social prescribing has existed in different forms, in a number of GP practices around the country for several decades [12, 13], but the recent national roll-out marks a significant expansion - NHS England has committed to hiring 1000 link workers across the UK over 2019/2020, with the aim for social prescribing to reach 900,000 people by 2023 [14].

There is emerging evidence that social prescribing activities can support people’s mental health, with activities such as arts classes, gardening, and exercise schemes leading to increased empowerment, self-esteem, confidence, improved mental health outcomes and cognitive functioning, and lowered feelings of social exclusion and isolation [15–17]. Another social prescribing study found reductions in isolation and improvements in health-related behaviours and management of long-term conditions [18]. There is also growing evidence for the benefits of the common social prescribing ‘model’, that is, the referral from a GP, through a link worker, to community groups and activities. For example, a randomised controlled trial of such a social prescribing model in Bristol demonstrated statistically significant improvements in anxiety, quality of life and ability to carry out daily activities [13]. A realist review of social prescribing in the UK has found that link workers form a crucial component of the model, facilitating the bridge between GPs and community groups, enabling greater access to support for patients [19].

Social prescribing is a complex system, with multiple interacting components, each activating different mechanisms, producing multiple and combined effects [20]. Therefore, it is vital to study the perspectives and outcomes of GPs, link workers, patients and community groups together. However, most evidence on social prescribing to date is from the perspective of the patient and their outcomes. Various studies have shown the benefits of the link worker role to patients, and some are also starting to evaluate social prescribing from the perspective of link workers [21, 22]. But the role of the GP in social prescribing is less well understood. Studies have identified that the success of social prescribing seems to rest on the GP’s ability to identify social issues and root cause [23]. It also appears to rely on GP “buy-in” to validate the service among other professionals and patients, and requires GPs to believe in the link worker’s ability and in the benefits of social prescribing [22]. Further, a few studies have included interviews with GPs, but these have tended to involve a very small number of GP interviews, or focused solely on 1 practice or locale [24, 25]. Nevertheless, this preliminary research does demonstrate that GPs found it challenging to have good knowledge of community groups or the time to engage fully, but valued face-to-face meetings with them [24]. GPs were also reported to find it difficult to address patient’s social and mental health needs, due to lack of training and limited time in appointments; GPs acknowledged the limitations of the “traditional medical model” [25]. And so, this stresses the importance of pursuing this line of inquiry, to understand the role of GPs in social prescribing more clearly.

Therefore, this study is the first to explore the barriers and enablers to social prescribing for patients with mental health problems, from the perspectives of GPs from across the UK. It uses the lens of behavioural change theory to examine this, applying the COM-B model [26]. The COM-B model is systematically derived from multiple existing behaviour change frameworks and finds that human behaviour is driven by a combination of Capability (having the physical and psychological skills to enact a certain behaviour), Opportunity (the physical, environmental and social circumstances in which a behaviour can be enacted) and Motivation (the reflective and autonomic mental processes involved in driving behaviour). This study uses this COM-B model to elucidate the barriers and enablers to GPs’ social prescribing and engagement with community groups, for patients with mental health problems.

## Methods

### 1Design

Interpretative-descriptive qualitative methods [27] using a one-to-one interviewing approach were used to understand what GPs experienced to be the barriers and enablers to engaging with social prescribing for patients with mental health problems. Telephone interviews were chosen since this was thought to be more convenient for professionals and allowed for a greater geographical spread of GPs. The one-to-one interview approach was chosen in order to allow time for in-depth analysis of individual GPs’ perspectives, without any peer influence and restrictions which might arise from focus groups.

### Participants and procedure

Seventeen GPs were interviewed, once each, with each interview lasting from 30 to 45 min, conducted over the phone.

Community groups and activities were defined as any group, service or activity within the community, often provided by the voluntary sector; not NHS services e.g. CAMHS. Examples were given such as arts groups, peer-support, walking clubs and community gardening, or anything understood by GPs as “social prescribing”. A purposive sampling approach was taken, to reflect potential differences in barriers and enablers due to GP age, gender, geographical region, known prior engagement with social prescribing, size of practice, and GP career level [28] (see Table 1 for characteristics of GPs). Recruitment took place through the mailing list of a national research network (the MARCH network), existing contacts of the lead researcher and university team, and a practitioners’ newsletter (the Social Prescribing Network). No monetary or other incentives were offered for participants to take part. The study received approval from the University College London (UCL) ethics committee (14,895/002) and all participants gave informed consent. A topic guide for conducting the interviews was developed using the COM-B model as a framework. This guide is presented in Supplementary Material. Interviews were recorded and then transcribed by transcription service ‘Way With Words’ in anonymous format.

**Table 1:** Characteristics of GPs

Region	Wales	1
	East of England	2
	West Midlands	1
	South West England	3
	South East England	1
	London	9
GP type	Partner	8
	Sessional – salaried, locum	6 (incl. 2 PCN Clinical Directors)
	Junior, trainee	3
Gender	Male	6
	Female	11

### Data analysis

The analytical approach used was reflexive thematic analysis [29]. This consisted of following the steps set out by Braun and Clarke [30]: familiarisation with the data, generation of initial codes and clear definition of codes, searching for themes, reviewing themes, defining and naming themes, and producing the report. Themes were verified with a second researcher (LB). The software used for coding was NVivo qualitative data analysis; QSR International Pty Ltd. Version 11, 2015. The analysis consisted of both inductive and deductive techniques: initial coding was conducted in an open manner, allowing codes and themes to be grounded within the data. The context around codes was retained and contradictory data was also included. Coding was undertaken at the semantic level, covering what has been explicitly articulated by participants, using theory to progress the level of analysis from description to interpretation, elucidating the barriers and enablers of behaviour within the COM-B model.

Codes were then grouped into themes. Each theme represents a “central organising concept” describing a meaningful pattern in the data [29], and falls within Capability, Opportunity or Motivation: the three domains of the COM-B model. We analysed our data in relation to the physical and psychological capabilities of individual GPs as reported by them, their reflective and autonomic motivations, and the social, environmental and physical opportunities available to them. Following analysis, we applied the Theoretical Domains Framework (TDF) [31]. The TDF was originally developed using an expert consensus method that synthesised, from a plethora of behaviour change theories, 14 key domains. These domains map onto, and add a greater level of depth to, the COM-B model. We then used a matrix that matches the theoretical domains to specific behaviour change techniques, based on expert consensus for effectiveness at behaviour change [32, 33]. This enables the mapping of specific barriers and enablers identified by the COM-B model to types of interventions that change behaviour. This allows us to identify and suggest interventions that may support GPs tackle the barriers and enablers to their engagement with social prescribing.

## Findings

### The participants

### Themes

**Fig. 1:**
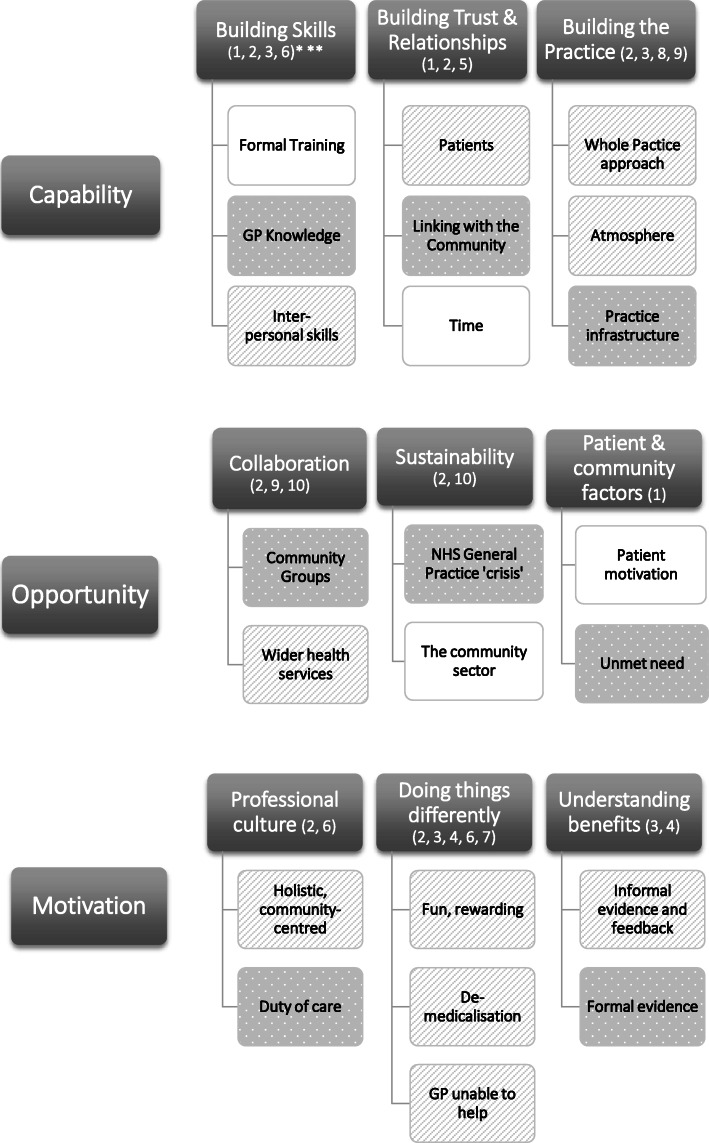
Themes, sub-themes and links to COM-B model. *Dark grey rectangle = Capabilities, Opportunities, Motivations; white rectangle = barrier; striped rectangle = enabler; dotted rectangle = both enabler and barrier. **The numbers in brackets refer to the intervention strategies proposed to address each sub-theme, see Table 2

Nine primary themes were identified. These were: building skills, building trust and relationships, building the practice, collaboration, sustainability, patient and community factors, professional culture, ‘doing things differently’ and understanding benefits. The sub-themes, and how these map onto the COM-B model, are displayed in Fig. 1**.**

#### Capability

Three themes were identified within the category of capability – ‘Building skills’, ‘Building trust and relationships’ and ‘Building the practice’.

##### Building skills

‘Building skills’ encompassed 3 sub-themes, which consisted of barriers, enablers and mixes of both – including a lack of formal training, GP knowledge of social prescribing evidence and of local community, and the benefits of GPs having a range of strong inter-personal skills when relating to patients.

### Formal training

Most GPs said that they received very little training in terms of how to engage with community groups to support social prescribing for patients with mental health problems. Most perceived the lack of formal training to be a barrier, as it meant they lacked the motivation, confidence and know-how to engage in social prescribing effectively:

*“As a GP for 30 years I am very conscious that there a lot of people that I cannot help with these skills and training that I’ve received so far as a doctor”* (GP6)

Some GPs felt that when they did have teaching related to this area, it was treated as somewhat of an ‘add-on’, secondary to the biomedical and clinical teaching:

*“it’s always thought of as a little asterisk…it gets thrown in on the side like oh, don’t forget about social prescribing”* (GP13, GP trainee)

GPs felt that there were significant differences in training on this between different GP practices. Part of this was linked to how well-established the level of community engagement was in that particular practice:

*“you’ve got one practice where it’s completely commonplace to do loads of social prescribing, and another practice where it might not be something they do at all”* (GP13, GP trainee)

It was felt that formal training at an early stage in a GPs’ career, would be beneficial. One GP trainee described how ‘sitting in’ with link workers and welfare officers to observe what they do, was a highly valuable learning experience:

*“At the beginning, when I started the job, I had a two-week induction, where every day I would sit in a different clinic, sit in a different community service… so that’s how I learnt about it”* (GP14, GP trainee)

### GP knowledge: evidence and local community

GPs talked about the importance of having the knowledge of evidence related to this area, both regarding the wider evidence base but also that specific to their own social prescribing scheme. GP knowledge of local community assets was deemed important but difficult for most GPs to obtain – part of this was related to the time constraints and demands of the job, which made it difficult for GPs to improve their knowledge of the local community:

*“it’s about not knowing what’s out there that can help us above what we’ve already got in our surgery…we’re always busy…We don’t always have enough time…”* (GP17)

GPs found it challenging to keep up with all the new services and groups regularly cropping up, often replacing old ones that had closed down, within the community:

*“it’s really difficult as a GP because things change so much and services are available and then they’re not available”* (GP4)

GP knowledge of local community groups and activities was enhanced by personal meetings between GPs and local community representatives, especially if they physically visited the practice:

*“what really works well is when a human being physically comes to our site to physically one-to-one reinforce the value of the service that’s on offer”* (GP3)

Further barriers to good knowledge of the local community were the large numbers of part-time and locum GPs, and high staff turnover. This meant that GPs have less time to build knowledge of the local community, and that new GPs who fill the vacancies, have to start afresh, too. It also meant that the sharing of knowledge and support between GPs is more difficult, as they share fewer overlapping shifts:

*“I work part-time and the other GPs in the practice work part-time. There isn’t a single time when we’re altogether at the same time, in order to be able to say, did you know this is happening?”* (GP1)

Many of the GPs had not themselves accessed the types of community groups and services that patients might be referred to. This may underplay these groups’ importance in the eyes of doctors, and also the extent to which they are known about:

*“Having just led a very sheltered, privileged life…I’ve got no idea about any of this stuff”* (GP15)

### Inter-personal skills

There were a range of important inter-personal skills of GPs that emerged from the interviews, including: taking a personalised approach, the importance of listening, perseverance of GPs, and having a proactive instead of reactive style.

GPs frequently talked about the importance of developing a personalised, patient-centred approach, based on what matters to the patient. This was necessary to find the ‘right fit’ for patients, thus enabling effective social prescribing:

*“For me, the biggest thing is more around what people want. Because it’s not my choice at the end of the day, it’s the patient and what they want, and what they think will be helpful to them.”* (GP12)

GPs ‘going the extra mile’ was commonplace, and often of fundamental importance for facilitating patient engagement with community groups. Part of this might involve GPs’ actions: visiting community groups, setting up community projects, or learning at conferences; and part of this relates to GPs’ inter-personal skills: their perseverance with patients and ‘sticking with it’ approach:

*“I’m actually interested in what matters to you. I’m not looking to confirm or refute a diagnosis, I’m actually looking to see if between you and me we can find a way, a route map, out of the place you’re in, to a place where you would rather be… stick with that person until they have made the necessary connections, applied the necessary advice…whatever it is”* (GP6)

GPs referred to the benefits of taking a proactive, over a reactive, approach to supporting patients with mental health problems engage with the community:

*“My knowledge of the community doesn’t come from any of the practice jobs that I’ve had. It comes from actually being interested, and being engaged, going to community meetings”* (GP1)

The importance of truly listening to patients, was stressed by numerous GPs. One GP spoke of how their practice gained a reputation for this within the community, showing the wider knock-on effects that are possible:

*“Number one, we listen… people were actually shocked and they said, “you just listened to me!”…thereby by reputation which is how news tend to travel in a deprived but close community, fairly rapidly started to attract those people that needed listening to”* (GP6)

Overlapping with both good listening and the ‘sticking with it’ approach, is the ability of GPs to understand the ‘root causes’ of patients’ problems. It was felt that the root cause for many patients’ mental health problems was largely social in origin, for example related to loneliness, financial difficulties, or lack of meaning or purpose in their lives. Thus, in order for GPs to know when, and how, to use social prescribing, it requires an enhanced knowledge and understanding of these wider patient circumstances.

*“when you see other mental health patients…especially on the milder scale, it’s evident that a lot of the problems that people are having are hugely tied into their social life and their social circumstances”* (GP13)

#### Building trust and relationships

The theme of ‘Building trust and relationships’ emerged between both the GP and patient, which was regarded as an enabler, and between the GP and the wider community, which was also valuable but considered challenging to develop by GPs – although was strongly facilitated when there was a link worker**.** Time was fundamental in building those trust-bound relationships, but was in short supply for GPs.

### Patients

Trust, from a patient towards a GP, was a recurring theme, crucial in enabling GPs to successfully motivate patients to engage with the community, or link worker. Part of this comes from GPs’ inter-personal skills. Building trust also takes time – some long-standing GPs had this, having spent considerable time with patients getting to know them well:

*“I know my patients really well. I’ve been there for 14 years so I’ve got a group of patients that I know very well, I’ve got a rapport with, I feel if I suggest something to them, they’ll generally think it might be a good idea, and try and follow through.”* (GP5)

### Linking with the community

Trust between GPs and the local community was also important. GPs had to trust in the community services that they were referring patients to. Part of this, again, was facilitated by GPs being in the practice for a long-time:

*“we’re a very long-standing practice… we know the local community… we are, in that sense, quite old-fashioned"* (GP3)

Most GPs had limited capacity to form trusting and working relationships with many community groups and services directly:

*“The issues would really, I think would be around relationships. So, I don’t think it’s possible for every single person to have good working relationships with every community organisation that’s out there. We’re just too constrained in terms of the people that we know”* (GP1)

The link worker’s role was therefore considered of fundamental importance in building the relationships with community groups, and enabling effective social prescribing:

*“I think that’s where the link worker comes in, because it’s about creating a bridge. You just have that one relationship with that one person, who has that relationship with multiple areas”* (GP1)

Again, regular, face-to-face connections worked most effectively to build the necessary relationships. The value of this was deemed difficult to measure, but considered highly important:

*“by coming fairly regularly, just refreshes us and makes us think about it…the value of a physical person is really hard to quantify, but it’s really valuable”* (GP3)

### Time

It takes time to develop the level of trust and working relationship required to motivate patients to take up a community activity, or meet with a link worker, and this was a significant barrier for participants. This ‘motivational threshold’ relies on both time within an appointment, and time spent over a longer period of weeks, months to years between doctor and patient:

*“as GPs we just don’t have the time. And I don’t have time to have a consultation with someone on loneliness and how they can deal with that”* (GP15)

*“that could last for 3,6,9,12 months, occasionally even longer to develop that sort of action plan… sometimes you have to get people down the road before they will accept a social prescribing referral and just hold them for a time until they trust that your referral is well intended and expertly suggested”* (GP6)

Again, the high levels of locum and part-time GPs, and high staff turnover rates, were barriers to building those highly time- and trust-dependent relationships with patients.

*“If you’re a new doctor or somebody that moves around without a consistent patient group, it is harder, I think to have that relationship with patients, particularly if you’re suggesting something a little bit different for them”* (GP5)

It also takes time for GPs to develop vital relationships with community groups, that they might refer patients to.

There is also a time-investment required from GPs to be creative, proactive, or go the extra mile, in engaging with the community:

*“if you’re trying to do anything that’s slightly different or creative…it requires time and resource”* (GP11)

#### Building the practice

‘Building the practice’ encompassed 3 sub-themes: taking a whole practice approach, building the practice atmosphere, and practice infrastructure (which encompassed digital and physical elements).

### Whole practice approach

GPs frequently articulated the benefits of taking a ‘whole practice’ approach to enabling effective social prescribing for patients with mental health problems. This could involve joint training, or meetings involving all practice staff members:

*“we’ve had the social prescriber presenting at one of these…telling people what her role is, what she thinks she can help with, how to refer to her, etc. And we communicate quite widely within the practice”* (GP5)

One GP talked about the ‘glue’ of the practice, often kept together by a number of key staff. In addition to the importance of partners, practice managers, link workers and patient groups, several GPs talked about tapping into the potential of receptionists, who had the skills to support patients, and often lived locally which positioned them well within the community:

*“Most of the reception staff that work in the practice around here, are local. And so, they know local residents, they know, to some extent, what you’d know in the community if you lived in that community. They would know the kind of services…”* (GP1)

GPs expressed the utility of having a link worker ‘in-house’, enabling personal, face-to-face and ongoing connections between them and practice staff. This facilitated more effective shared learning, regular feedback, and helped GPs understand the social prescribing service and referral criteria. The link worker provides a regular reminder that social prescribing is a tool at the GP’s disposal, for patients with mental health problems:

*“I’m very lucky so I pop in and see her maybe once a week or so…I think it’s really helpful for the doctors and nurses who work in our building, with her [the link worker]…keeping everybody enthused about the project, remembering that she’s there and we should be using her to refer patients or getting feedback from her about people who have successfully engaged and feel better. It makes it more real”* (GP5)

Relating to the whole-practice approach, but also running through all the capability themes and sub-themes, it emerged that many GPs considered there to be striking difference between individual GPs regarding their skills, motivation and knowledge in this area.

*“each individual clinician will have different knowledge of the community, and a different attitude and approach towards linking people in with other resources in the community. That will come down to individual clinical practice, as opposed to a specific practice policy”* (GP1)

### Atmosphere

It was a key enabler when a GP’s practice had an atmosphere of community-centredness. The atmosphere of the practice is closely linked to the practice culture, which will be discussed later in the category of motivation, but here refers more to the structural elements of the practice which helps create a ‘feel’ of it being welcoming, and centred around the community. A welcoming practice made it easier for GPs to engage patients with community-centred approaches. Practices that ‘invited patients in’, who then themselves set up activities based within the practice, reflected this approach.

‘Knock-on effects’ on the wider community emerged from having a community-centred atmosphere. For example, in one practice, that had a community garden within it:

*“a couple of policemen came by, and at first I thought, oh dear, maybe there’s been some vandalism…but it wasn’t that at all. They had heard about the community garden…they had some young offender in mind who they thought was just bored, and might benefit from actually doing something on the land. They were coming to chat…to see what was possible”* (GP17)

Further ‘knock-on effects’ were seen within a practice that embedded a weekly arts & crafts session in their waiting room. The perception of the practice changed in the eyes of patients, who saw it ‘in a different way’ and more welcoming.

*“Many less complaints. Patients are, generally, nicer at the desk…some of our patients we know come to the crafting group because they’re sitting in our waiting room and they see what our receptionist has to deal with…they see them in a different way. So, I think it’s broken down some of those barriers and put a more human face on the practice”* (GP11)

### Practice infrastructure

A further sub-theme was around the infrastructure of the practice, encompassing both digital and physical infrastructure. When good Information Technology (IT) systems were in place, this allowed easy referrals from GPs to community groups or a link worker, which made a GP’s job much easier in referring patients with mental health problems for community support.

For example, one practice utilised a single database that was used by the local Clinical Commissioning Group (CCG), used for social prescribing referrals, alongside clinical referrals such as cardiology appointments or hearing tests:

*“We’re quite helped by the fact that we have one database of referral forms…all our referral forms are uploaded onto that…So that’s become easier to integrate new services, because of the IT really…”* (GP3)

The issue of lack of physical space also emerged with several GPs:

*“I’d like more room physically… If we had more space we could invite the community and the link workers in more closely, which would be an advantage”* (GP6)

The practice space could also be used more effectively to advertise social prescribing, for example in the waiting area:

*“probably wasn’t advertised well in the waiting area, the areas that the patients stand at the reception desk, so I think that probably could help”* (GP14)

#### Opportunity

Three themes were identified within the category of opportunity – ‘Collaboration’, ‘Sustainability’, and ‘Patient and community factors’.

### Collaboration

‘Collaboration’ encompassed 2 sub-themes, consisting of GP collaboration with community groups, and GP collaboration with wider health services.

### Community groups

Ongoing collaboration between GPs, practices and community groups was vital for successful social prescribing. This was, in part, mediated by the informal relationships previously discussed. More formalised collaborations were also important – a common and highly effective example of this was through the use of a link worker:

*“she (the link worker) was a brilliant point of contact just to get plugged into that side of things. Because to be honest, before GP I was completely oblivious to all this stuff” (GP15)*

As mentioned previously, GP practices can also collaborate directly with the community, for example citizens being invited in to set up groups, activities and events within the practice. Some GPs felt that the formalisation of collaboration was useful, as it meant it was more likely to be sustained longer term:

*“we often have conversations about oh, it would be great to do this, this and this…The problem is, it’s too ad hoc, this is more about formalising it and having an actual program…Because I think unless you get that in, it’s difficult to sustain it just by people’s good intentions and motivation and things”* (GP9)

### Wider health services

GPs also articulated the benefits of formally collaborating with wider health and care services, to facilitate more effective social prescribing. They spoke of the importance of the newly formed Primary Care Networks (PCNs) to aid this. Working more closely with neighbouring GP practices enabled more efficient pooling of resources, sharing of knowledge and greater community support for patients. This was especially felt by small practices:

*“We’ve always been motivated in principle, but we really didn’t have the wherewithal, especially being a small practice, to set it up ourselves effectively…it’s really been the advent of being part of a primary care network that’s changed the landscape for us”* (GP3)

Asked what factors enable successful social prescribing, GPs also talked about the importance of collaboration at the CCG (Clinical Commissioning Group)-level, and local authority level. CCGs could target resources to support community engagement effectively:

*“if it’s CCG-wide. If the CCG sources say, right, we are paying for this service for our patients, that’s brilliant”* (GP2)

*“I think it’s about making the case for robust community investment for intelligent and authentic social prescribing link worker activity for building primary care networks into their local strategic partnership committees, their local authority conversations”* (GP6)

#### Sustainability

‘Sustainability’ included 2 sub-themes – the ‘crisis’ in the NHS and General Practice, and the sustainability of the community sector.

### NHS and general practice ‘crisis’

GPs mentioned resource pressures that affected the degree to which they could effectively engage with community groups, on behalf of patients with mental health problems. However, counter-intuitively, perhaps because of that strain, there was acknowledgement from GPs that they needed to engage with the community and third sector, in order to help those patients:

*“the NHS is under strain, there’s not enough appointments, not enough time, not enough doctors, not enough nurses, it’s just very difficult when you’re trying to survive to be able to support as you would want to”* (GP4)

“*I think the social prescribing and the community activities, like Men in Sheds, and other things, have really met some of that need”* (GP7)

### The community sector

The sustainability and funding precarity of VCS (Voluntary and Community Sector) organisations, was well understood among GPs. Their concern was related to future sustainability and availability of these organisations, which in part was based on GPs’ experience of groups disappearing – this made it challenging to keep up with what is available, and also form lasting relationships with community groups:

*“There’s no point just having somebody signposting if there’s nothing there to signpost them to…There used to be quite a number of community groups going…. there’s very little activity they do now there…They’re all gone. There’s nothing really available”* (GP16)

Patients might become reliant on community groups or activities for their health and well-being, and so there was also concern from GPs about the time-bound nature of certain community activities and projects:

*“they would have some support, but it would finish after the prescribed amount of time. So, I had one patient who was invited to a gardening project, he was given 16 sessions. And actually, ended up in hospital when that provision was taken away because I think that the contrast between having activity and having some social support and then having it removed was almost worse, for him, than having nothing at the beginning”* (GP11)

Linking to the earlier theme of formal collaboration, one GP suggested an opportunity for greater community support through the shift from Sustainability Transformation Partnerships (STPs) to Integrated Care systems (ICSs):

*“We’ve gone to the STP and have said to them if you become an ICS, you really need to think about how you are going to attract funding into the third sector of the communities”* (GP6)

#### Patient and community factors

‘Patient and community factors’ also contained 2 sub-themes which referred to patient motivation and high levels of unmet, psychosocial needs.

### Patient motivation

GPs felt that the patient’s own motivation or willingness to engage with social prescribing, was often a crucial barrier. GPs found it challenging to persuade some patients to see a link worker, or try a community activity. Beyond the initial engagement, there is also the issue of more longer-term adherence. Patients with common mental health problems such as anxiety and depression find it particularly difficult to try something new, especially when they are unwell:

*“There are real issues around motivation, effort, concentration, decision-making particularly people who are anxious, to go and try something new”* (GP12)

There seems to be a ‘motivational threshold’ that patients have to surpass in order to agree to engage with social prescribing, then actually turning up for a group or activity, and then continuing to show up:

*“one of my concerns is around how to help patients get over the threshold, so, the threshold in terms of actually signing up and the threshold of actually joining the group”* (GP16)

When groups were labelled as being ‘for mental health’, ‘for social isolation’, or something similar, this was often seen as a barrier to persuading patients to attend. GPs felt it was more effective to focus on the activity itself, and whether it was something the patient might enjoy:

*“I think one of the biggest barriers is anything they perceive as being specific to people with mental health problems. So any kind of activity that’s labelled as being for lonely people… it has to be much more around…what they’re doing, and how interested they are in the activity itself”* (GP12)

Accessibility of, and transport to, community groups for patients was often a key barrier. This could be due to poor transport links in the area, the cost of transport, or patient aversion to travelling far from home:

*“We have a barrier of accessibility. So many of these people don’t have any means of transport, aren’t confident enough or able to use public transport”* (GP5)

GPs found that some patients required extra support to engage with community groups. This might require someone meeting them face-to-face, perhaps even accompanying them to the first session. Again, for those GPs that had access to one, the role of the link worker was a crucial enabler in helping patients engage, bridging that gap between the GP and community, where patients often struggle to navigate alone:

*“They need help with getting to appointments…it’s almost like a hand holding role…And this is really critical, what I find with a lot of our mental health patients is that you can tell them to go to this service…But actually the gap between the GP and actually getting there is where we lose them so often, and that is where the navigator is really key”* (GP9)

### Unmet need

Most GPs in this study saw high levels of unmet, social needs within their patient population – and many felt these levels had been rising in recent years. Because of these largely social needs, it was clear to most participants that a social solution, rather than a purely clinical or biomedical one, was more likely to be effective. GPs saw social prescribing, and engagement with community groups, as a key tool especially for patients with mild or moderate mental health problems:

*“the answer probably needs to come from the community because that’s where the problem started”* (GP10)

This is most pronounced in deprived areas, demonstrating the social gradient of mental health problems, reinforcing inequalities:

*“housing, financials, benefit stuff, debt, employment issues. They’re the biggest things coming into practices… a lot of people that I see, 70% I’d estimate…are coming in with…mental unhappiness, lack of mental well-being… That kind of stuff is what I think is common in deprived areas…and it places a huge burden on practices”* (GP1)

#### Motivation

The category of motivation contained 3 sub-themes: ‘Professional culture, ‘Doing things differently’ and ‘Understanding benefits’.

### Professional culture

‘Professional culture’ encompassed two sub-themes: holistic, community-centred care and duty of care.

### Holistic, community-centred: care

Holistic and community-centred approaches often formed part of professionals’ and practices’ culture and ethos, which enabled GPs more extensive social prescribing. Being a long-standing practice with long-serving GPs often enhanced this. Individual professional ethos or culture both influenced and was influenced by the overall practice culture:

*“it plays to our philosophy of trying to offer holistic care… three of us Partners have been here for the better part of 25 to 30 years… We know multi-generations within the same families, know the local community…we’re quite embedded in the community…makes us better able to integrate and persuade people to go and use other community services.”* (GP3)

However, it was often the case that GPs felt their practice culture was not firmly rooted in community. Part of this was due to individual GPs having no connection to the local area:

*“I don’t see any of the practices in this geography as being really rooted as community organisations. So, certainly there are practice staff who’ve been here for decades who have never walked around this area. They drive to work, and they’ll drive away from work and don’t live locally.”* (GP1)

### Duty of care

GPs felt passionately about the principle of ‘duty of care’, and were driven by this and the principle of providing a high-quality service of care for their patients. When community engagement, or social prescribing, was considering a component of ‘high-quality’ care, it was a key motivating factor:

*“I think it’s a wish for a high quality service for patients”* (GP7)

*“There is a duty of care to these patients…one of the main things I can do for any patient is to signpost them to the available resources”* (GP2)

Part of it being high quality care stems from GPs’ belief that social prescribing is effective, which ties into the later sub-theme of ‘Understanding benefits’ and the importance of feedback and evidence.

#### Doing things differently

‘Doing things differently’ referred to social prescribing being fun and rewarding for GPs, the desire to de-medicalise, and the GP-felt inability to help with social issues.

### Fun, rewarding

Some GPs talked about how it was fun to actively engage more with community groups, and that they found this process rewarding. Linked to the earlier point about whether this is ‘part of the role’ for a GP, there is a tension around professional boundaries, that requires overcoming:

*“It’s actually fun to find novel and creative ways to help your patients much more than prescribing a statin or an anti-depressant. Although it does require a little bit of breaking down boundaries…So, there’s a certain inherent tension there”* (GP10)

One GP, whose practice invited citizens from the community in to run groups within the practice, such as a weekly crafting session, expressed that this creative and ‘different’ process was rewarding for practice staff:

*“because it’s all been voluntary in a way, and that, actually I think it has engaged the staff group because, again, I think they have quite enjoyed seeing different things happening around the practice”* (GP11)

GPs were fundamentally driven by a drive to help patients, and make them feel better – which was a key enabler because it was felt that social prescribing could offer that:

*“I love medicine, but fundamentally, I like making people feel better, and there is obviously a lot of social stuff that comes into play here”* (GP15)

GPs also spoke of the desire to ‘empower’ patients, so they can take control of their own lives and health:

*“I’ve always been interested in the idea of empowering patients to take charge and control of their own conditions and managing their own conditions”* (GP17)

The ‘fun’ and ‘rewarding’ components are perhaps especially important given the current high levels of stress and burnout among GPs:

*“all around people are burning out, in the last 3 years we’ve had 6 salary GPs leave and each one has cited this intensity as being the reason why”* (GP10)

### Desire to de-medicalise

GPs talked about the need to de-medicalise certain patient problems that they felt had been over-medicalised. GPs understood many patients’ mental health problems were influenced or caused by their social circumstances, for example related to social isolation, housing or financial difficulties. There was a desire among GPs to look for social solutions for these patients, whose problems were rooted in their social circumstances:

*“there’s a massive role for the community in promoting…mental health and well-being (because) actually most of the mental health and well-being has got social causes”* (GP10)

*“a large proportion of our people who attend frequently, who are often struggling with chronic pain, struggling with chronic mental health issues and have social isolation…Many of these conditions are not really amenable to medicalisation”* (GP5)

The desire to de-medicalise is closely influenced by the previous sub-theme of inter-personal skills, especially the ability of GPs to understand the ‘root causes’ of patients’ issues – which were often primarily social, not medical.

### GP unable to help

GPs often felt unable to help patients with psychosocial issues, with the tools at their disposal – that is, with both their professional skills and the medication they prescribe. GPs felt social prescribing provided such a tool, helping meet those patient needs for which GPs felt they could support no further on their own:

*“a lot of the problems that people are having hugely ties into their social life and their social circumstances…there’s nothing that I can personally do to help that. And you think if only you could get out and do a walking group, do an art class, do something, that would help with a lot of your issues”* (GP12)

There was a common belief in the limitations of certain medications, especially anti-depressants, as the primary solution for patients with mild or moderate mental health problems:

*“we medicalise unhappiness as depression…but does that mean they actually are depressed? They get medicalised, get given anti-depressants and get given neuropathic drugs, benzos and opioids. Whereas in fact, when you drill down to it, it’s because they’ve got no hope and no control and no agency. It’s because they feel valueless, all the sorts of reasons which those drugs will never treat”* (GP1)

#### Understanding benefits

‘Understanding benefits’ included both informal and formal evidence of social prescribing.

### Informal evidence and feedback

It was very rewarding for GPs to feel that patients were benefiting from social prescribing. An effective way of fulfilling this need was having regular feedback to the GP of how the patient was getting on, after their initial referral. GPs, driven by a desire for high quality care for their patients, were far more likely to continually engage in social prescribing, if they knew their patients were benefiting. This process was made easier with a link worker, especially if they were based in the practice building.

*“it is important that we get feedback and we understand what’s happening. We’re lucky, she’s based in the same building and we speak with her frequently”* (GP5)

Another way, other than regular feedback, of GPs knowing this can benefit patients, was through GPs’ personal experiences. As discussed previously, doctors might not be as likely to have accessed those community resources as some of their patients, but when they were able to relate it was highly motivating:

*“So, I was ill myself probably about seven or eight years ago. And at that time I was struggling to work so what I did was I went to an art class…And I think certainly for patients of mine with mental health problems or even actually things like chronic pain or breathlessness or any of those things being able to focus on an activity I think is really helpful for them”* (GP16)

### Formal evidence

Alongside the importance of feedback and informal evidence, GPs talked about the importance of formal evidence demonstrating that social prescribing was effective. This includes both wider research, as well as research conducted on their own practice’s social prescribing model and patient population. GPs were far more likely to use social prescribing if they had a strong evidence base that it improved patient outcomes:

*“We want to try and have some evidence to prove that patients are benefiting, so that we can go on employing somebody in this role and applying for funding and stuff”* (GP5)

Some of the difficulties conducting this sort of research is due to research getting in the way of the activities, and some of it relates to the fatigue of third sector organisations and clients having to fill in continuous tick boxes and questionnaires.

*“it’s like the art therapy, we know it’s making a huge difference and we can do surveys or different things, but does it really capture that it’s actually reducing, improving well-being? Those kind of things, without stifling the organisations, or the patients with survey after survey, or questionnaire”* (GP9)

## Discussion

This study explored the barriers and enablers to social prescribing for patients with mental health problems, from the perspectives of GPs. Most GPs were supportive of social prescribing and active engagement with community groups, with nearly all the themes within motivation being enablers. For example, GPs were motivated by a desire to move away from the status quo in primary care, which they felt was failing many patients and leaving them with unmet, psychosocial needs. This was coupled with efforts to de-medicalise social problems amongst patients and find alternatives where medications were found to be ineffective, corroborating wider research [8]. It was often enjoyable and rewarding for GPs to support this work, which, given the current high levels of GP burnout and stress [34], has positive implications for staff well-being, morale and GP retention. There were a range of inter-personal skills that GPs felt were important to successfully engage, including active listening, ‘sticking with it’, taking a personalised approach with patients, and the ability to get to the ‘root causes’ of patients’ problems. Trust was also fundamental - patients had to trust GPs before they could overcome the ‘motivational threshold’ of agreeing to see a link worker, or attend a community activity. This is consistent with research demonstrating that patients who have high levels of trust in their doctor are significantly more likely to adhere to the healthy behaviours the doctor recommends [35, 36].

Further, although GPs felt very limited by the 10 min appointments they had with patients in building this trust, they believed that link workers (who often have ~ 1 h consultations) had the time to support patients with a more personalised approach. Link workers were also seen as the key ‘bridge’ between the GP and community, where previously GPs were limited by the number of relationships they could build with the different community groups. Time, trust and building relationships must all be seen within the conceptualisation of social prescribing as a complex system [37], with trust between different stakeholders (e.g. patients, GPs, link workers, and community groups) important at each different stage of the social prescribing pathway [20]. The importance of taking a whole-practice approach also embraces complexity, harnessing the potential of receptionists, practice managers, link workers, GP trainees and partners to help build a practice ethos and atmosphere that is centred around the community.

There were also a number of key barriers. In the wider environment, GPs were concerned about the availability of community groups in their surrounding area and their often transient nature, and understood that the precarity of funding for third sector groups was a significant challenge. GPs also spoke of the ‘crisis’ across the NHS and General Practice, citing lack of resources, time and staff shortages. This contributed to GP stress and burnout but also, inadvertently, helped GPs understand that the community sector could offer support that they themselves could not alone. Another key concern was around a lack of formal evidence on the benefits of social prescribing, both in terms of the wider evidence base and also that collated within a specific practice’s social prescribing model. There is growing evidence that social prescribing has the potential to improve mental health and well-being outcomes for patients [38–40], but this evidence appears not to be reaching GPs. This is consistent with the fact that most GPs felt there was very little formal training on community engagement and social prescribing. When ‘informal evidence’ was present, via regular feedback from the community or link worker to the GP, this provided a significant incentive for GPs to continually engage with social prescribing for their patients. Corroborating wider research, this seemed to be especially effective when there was positive feedback and reinforcement either from patients or link workers [41–43].

It is evident, therefore, that in order to tackle the barriers and amplify the enablers found in this study, interventions are needed. These have the potential to support GPs engage more effectively with community groups, for patients with mental health problems. Mapping the barriers and enablers onto the COM-B wheel, elucidates several types of intervention that could help GPs engage more effectively, and optimise social prescribing especially for patients with mental health problems [26]. Specific Behaviour Change techniques have been selected, based on the degree of available evidence supporting their efficacy for that type of barrier or enabler [33]. The proposed interventions derived from the data in this study are listed in Table 2, below:

**Table 2:** Proposed interventions to promote increased GP social prescribing and community engagement for patients with mental health problems, linked to Behaviour Change Techniques

Intervention number	Number of barriers/enablers that could be addressed	Intervention type	Behaviour Change techniques	Outline of strategy
**1**	5	Training	Behavioural rehearsal; demonstration of behaviour; instruction on how to perform a behaviour; problem solving; goal setting (behaviour and outcome); discrepancy between current behaviour and goal; social support; verbal persuasion about capability; monitoring of (outcomes of) behaviour	Training package for GPs to develop the appropriate inter-personal skills important for effective social prescribing for their patients, including motivational skills, active listening, perseverance and resilience training, ascertaining ‘root causes’; include regular training updates and “check-ins” for GPs; include clear training for GPs on referral criteria for social prescribing
**2**	4	Training; enablement	Self-monitoring of (outcomes of) behaviour; Goal setting (behaviour); goal setting (outcome); action planning; identity associated with changed behaviour; restructuring the social environment	Training programme/plan for whole practice and all practice staff – on how to create a more community-centred atmosphere and engage more with the community, as a practice. This should include more time allocated to supporting active engagement with the community; regular whole-practice meetings, activities and team-building exercises that are centred around community approaches/social prescribing. This should also include tailored support for part-time and locum GPs, and a plan for GP retention
**3**	3	Education	Information about social and environmental consequences; information about health consequences; information about antecedents; credible source; pros and cons	Educate all GPs on the wider evidence base of social prescribing, the harms of over-prescribing and over-medicalisation; enhance GPs’ and other practice staff’s knowledge of the local community assets and services on offer – this could be run by community group representatives or link workers; also include education about the social prescribing service and the new link worker role
**4**	3	Incentive; Enablement	Feedback on behaviour; feedback on outcomes of behaviour; positive reinforcement; social comparison; reward; identity associated with changed behaviour	Develop a system to provide regular, systematic (positive) feedback to GPs on their social prescribing-referred patients; reward GPs who use social prescribing effectively and appropriately
**5**	2	Enablement	Social support (emotional and practical)	Set up a buddy system for patients accompanying them to community groups and activities; use link workers and harness volunteers for this
**6**	2	Persuasion; modelling; training	Salience of consequences; information about emotional consequences; pros and cons; material incentives; comparative imagining of future outcomes; framing/reframing; credible source; identity associated with changed behaviour	Use patient stories, community group and GP experiences to persuade GPs that social prescribing/community engagement is an effective way to support patients; through use of videos and in-person accounts
**7**	1	Restriction; Coercion	Behaviour substitution; habit formation; habit reversal; punishment; social comparison	Dis-incentivise GPs for inappropriate anti-depressant/medical prescribing and not offering social prescribing when referral criteria are met
**8**	2	Enablement; Environmental restructuring	Prompt/queues; behavioural substitution; behavioural cueing; habit formation; habit reversal; social comparison; conserving mental resources; restructuring the physical environment	Develop a strong IT system for social prescribing referrals; use on-screen prompts for GPs to see the social prescribing option for every consultation (or every relevant consultation as determined by referral criteria); design quick, simple forms that GPs can send to the link worker
**9**	2	Environmental restructuring	Restructuring the physical environment; restructuring the social environment; adding objects to the environment	Physical space in practice re-purposed or created in order to house a link worker and/or for receptionists to have chat with patients, and/or to invite the community in to utilise, e.g. crafting session or community garden
**10**	1	Environmental structuring	Restructuring the physical and social environment	Provision of long-term funding to VCS groups that are receiving social prescribing referrals; explore pooled budgets, e.g. combined health, local government and third sector funding

### Limitations

This study had a number of strengths, including its good spread of rural and urban perspectives and participants from practices in diverse areas of differing levels of deprivation, and the involvement of GPs from across the full spectrum of career level. Further, the research was guided by an established theoretical framework and our use of multiple one-to-one interviews enabled us to confirm and explore themes in depth. However, there were some limitations. The study involved interviews with 17 GPs, which limits the generalisability of its findings. Moreover, it was surprising that some issues seemingly highly related to those with mental health problems, e.g. risk and safety considerations in referring patients to community groups and availability of trained staff in those settings, was not talked about frequently by the GPs, and further research should explore this. However, it is nonetheless the first study from the perspectives of GPs across the UK, in the social prescribing literature. GPs were interviewed from across the UK, but the majority were still from England. Given slight differences in the roll-out of schemes within Scotland, Wales and Northern Ireland, research into the potential effects of interventions will need to be adapted to local settings. The self-selection of participants also means that some GPs facing more extreme barriers to engagement (e.g. due to lack of time or awareness) may not have been able to take part. However, our study did include a number of participants with no current engagement in social prescribing, so it was not just limited to those who already were significantly involved.

## Conclusion

This study is the first to explore the barriers and enablers to social prescribing for patients with mental health problems, from the perspectives of GPs from across the UK. It highlights the need to address barriers such as lack of formal training for GPs on how to engage effectively, the importance of a range of strong inter-personal skills, and the benefits of the link worker role. Further studies are encouraged in order to test the effectiveness of the behaviour change interventions proposed. They should also examine the factors which affect uptake and long-term adherence of social prescribing by patients. Other qualitative methods, such as ethnography, could be deployed to examine social prescribing in greater depth.

## Supplementary information

**Additional file 1.** Interview guide attached.

## Data Availability

The datasets used and analysed during the current study are available from the corresponding authors on reasonable request.

## Abbreviations

BCT: Behaviour Change Theory
CCG: Clinical Commissioning Group
COM-B: Capability, Opportunity, Motivation and Behaviour
GP: General Practitioner
IAPT: Improving Access to Psychological Therapies
ICS: Integrated Care System
IT: Information Technology
NHS: National Health Service
PCN: Primary Care Network
STP: Sustainability Transformation Partnership
TDF: Theoretical Domains Framework
UK: United Kingdom
UCL: University College London
UCLH: University College London Hospital
VCS: Voluntary and Community Sector
