# Supplementary material for: Social prescribing for individuals with mental health problems: a qualitative study of barriers and enablers experienced by general practitioners
Source: BMC Fam Pract. 2020 Sep 21;21:194. doi: 10.1186/s12875-020-01264-0 (PMC7507286; doi:10.1186/s12875-020-01264-0)
Supplement: Supplementary file 1 — Additional file 1. Interview guide attached. [file 12875_2020_1264_MOESM1_ESM.docx]

GP group [telephone interviews]

Opening points

- We’ve invited you to be part of the study as the MARCH Network **(a research network focusing on social, cultural and community assets and mental health)** is conducting a research project trying to understand what factors might help people with mild or moderate mental health conditions to participate more in community activities, and/or what aspects might be preventing this. As part of this we are asking how policymakers, funders and commissioners engage with people with lived experience of mental illness and community activities.
- **Community activities and organisations include (but are by no means limited by): singing groups, walking groups, art classes, community gardening, anything considered “Social Prescribing”, or that relates to social and cultural assets within the community. This research is not about community health services e.g. CAMHS, district nurses or community physiotherapy - but do still mention anything you feel is relevant.**
- We are also separately asking mental health service users, and cultural and community organisations for their views.
- Some people that we talk to may be very involved, whereas others are may not have done so much, but we’re interested in all views.
- You’ve all had an information sheet about the research. The key things are that we are audio recording today’s interview, but your views today will be confidential and anonymised. So anything you say where you reference a person or your organisation we’ll be anonymising that. So you can speak freely.
- We’re holding several other interviews over the next few months to hear from more organisations and will be bringing together everything we learn from all of the groups in a final report that we can circulate to you.
- I have a list of core questions I’d like to ask and some prompts but do feel free to add any other thoughts too
- A final thing to say is that we’re only managing to speak to a select group of policymakers, commissioners and funders for this research, but if, when you’re answering questions, you are aware of different experiences from other organisations you’re connected to, please do share them too – anonymously is fine - as we want to try and get a rich picture of the experiences of lots of different organisations.

Opening question

1. Understanding the role in at the moment – what role are you in now? How does this (potentially) relate to cultural/ community engagement and people with lived experience of mental illness?
2. **How is your organisation currently supporting social prescribing, and/or the engagement of people with lived experience of mental illness in community activities? [make clear that the following questions relate to the work/ role, rather than personally]**

Three main questions

1. **What was it that first MOTIVATED you (i.e. your organisation) to support this area of work? What is it that still motivates you to support it?**
   1. *Was this included in your organisational strategies or government policies?*
   2. *Was there any kind of economic motivation like cost savings?*
   3. *Have you been influenced by the increasing discussions on mental health?*
   4. *Did affiliated organisations request it?*
   5. *Is it important for your organisation to do this/ part of the organisation identity?*
   6. *Did you see other organisations delivering similar work either in the UK or in other countries?*
2. **What factors, skills or characteristics that you think your organisation has in order to be able work in this area? What makes it able to engage/ successful in engaging?**

**If not working in this area, what do you think it is about your organisation that would need to change?**

- 1. *Do you feel your staff have the training needed to be able to support this kind of work?*
  2. *Are there any factors within your organisation that make it hard for you to engage with this kind of work at the moment?*
  3. *Do you have sufficient resources?*
  4. *Do you have good links with healthcare/mental healthcare OR community organisations [DELETE AS APPROPRIATE]?*
  5. *Are there any issues around strategy/funding/GDPR etc?*
  6. *How confident are you about working in this area? What would change/ improve this?*
  7. *What kind of support would help your organisation?*

1. **Are there factors in the wider environment (outside the immediate organisation) that help with, or hinder, this work? For example:**
   1. *Have you received specific funding that has allowed you to develop this work?*
   2. *Have you received support from other organisations/ funders/partners?*

Closing questions

1. [Speaking about your department/ organisation] Now you’ve started working in this space, do you think you will ever stop? Why/why not? OR If you’re not already engaged, what do you think might change that and make you want to engage?
